# Assessment of Continuous Pulse Oximetry Monitoring in Infants With Bronchiolitis in the Pediatric Emergency Department: A Cross-Sectional Study

**DOI:** 10.7759/cureus.74164

**Published:** 2024-11-21

**Authors:** Katie Gardner, Tiffany Curl, Katrina F Hurley

**Affiliations:** 1 Emergency Medicine, Izaak Walton Killam (IWK) Health, Halifax, CAN

**Keywords:** acute bronchiolitis, continuous monitoring, patient safety, pediatric emergency medicine, quality improvement research

## Abstract

Purpose

Bronchiolitis guidelines recommend intermittent pulse oximetry monitoring for stable infants. Continuous pulse oximetry can lead to harm for some infants with bronchiolitis but is still frequently used in emergency departments (EDs) for infants who do not require oxygen supplementation. Measuring continuous pulse oximetry use from medical charts can be challenging. This study aimed to (1) develop a feasible method for documenting the use of continuous pulse oximetry in infants with bronchiolitis and (2) explore factors influencing its use in this population.

Methods

We conducted a cross-sectional observational study in a tertiary pediatric ED. Infants 60 days and 12 months old with possible bronchiolitis and not requiring oxygen were included. The patients were recruited from January 11 to March 21, 2023. Research assistants directly observed the use of pulse oximetry monitors. The primary outcome was the proportion of infants placed on continuous pulse oximetry. The secondary outcomes included disease severity, admission, unplanned return visits, the use of supplemental oxygen, and the need for investigations and interventions.

Results

Eighteen infants were included in this study, with six (33.3%) placed on continuous pulse oximetry. The median disease severity score was similar between infants who were continuously monitored (6.5 (IQR 5.3, 10.8)) and those in the intermittent pulse oximetry group (4.5 (IQR 2, 9)). Infants in the continuous pulse oximetry group underwent more investigations and interventions than those in the intermittent pulse oximetry group: chest radiograph and bloodwork in 50% versus 8.3%, antibiotics in 33.3% versus 8.3%, bronchodilators in 33.3% versus 16.7%, and steroids in 16.7% versus 0%. In the continuous pulse oximetry group, four infants (66.7%), all of whom were placed on supplemental oxygen, were admitted, compared to one infant (8.3%) in the intermittent pulse oximetry group.

Conclusion

The decision to use continuous pulse oximetry may be influenced by provider characteristics. Objective parameters should be developed to guide its application and minimize potential harms associated with it.

## Introduction

Bronchiolitis, a common viral illness in infants, is a leading cause of morbidity and the primary reason for hospital admissions among children aged 0-4 in Canada [1]. While infants with bronchiolitis frequently experience oxygen desaturations, these events alone do not necessarily correlate with disease severity or the need for interventions [2]. In a study by Schuh et al., infants with bronchiolitis were less likely to be admitted when their oxygen saturation was artificially displayed as three points higher than their actual saturation, with no significant difference in unplanned return visits, supplemental oxygen use, or delayed hospitalization [3]. National guidelines recommend intermittent rather than continuous pulse oximetry for stable infants with bronchiolitis and an oxygen saturation of 90% or greater [4]. Over-reliance on continuous pulse oximetry may reduce the frequency of hands-on assessments and contribute to alarm fatigue [5]. There have been several quality improvement initiatives aimed at reducing the use of continuous pulse oximetry in hospitalized infants [6]. The prevalence of continuous pulse oximetry use for bronchiolitis in the pediatric emergency department (ED) has not been previously studied. 

Our objectives were to develop a feasible method for documenting the use of continuous pulse oximetry in the ED and understand the factors influencing its use among infants with bronchiolitis. This work is intended to provide baseline data for future quality improvement initiatives.

## Materials and methods

Context

We conducted a cross-sectional observational study in the pediatric ED at IWK Health, Halifax, Nova Scotia, Canada, which has an annual census of approximately 40,000. Registered nurses are responsible for determining which patients require continuous pulse oximetry or cardiorespiratory monitoring prior to physician assessment. There are no specific criteria for the use of continuous pulse oximetry.

Approach

Patients were recruited from January 11 to March 21, 2023, to align with the typical peak season for bronchiolitis in our community. A convenience sample was collected between 10 am and 10 pm, seven days a week, based on the availability of research assistants. We included infants aged 60 days to 12 months who presented for the first time with possible bronchiolitis (defined as the first episode of respiratory distress with cough, coryza, wheezing or crackles, and tachypnea or chest retractions) and had an oxygen saturation of 90% or greater at triage. Exclusion criteria included a history of complex chronic illness (cardiopulmonary disease, immunodeficiency, neuromuscular disease, or airway abnormalities), need for supplemental oxygen upon arrival, previously documented use of bronchodilators, or assignment of Canadian Triage and Acuity Scale (CTAS) level 1 at initial assessment. 

Research assistants performed repeated observations on an hourly basis. When patients met the study criteria, the research assistant recorded whether they were on continuous pulse oximetry and determined their Respiratory Distress Assessment Instrument (RDAI) score. To assess the RDAI, the research assistant interviewed the nurse caring for the patient and obtained verbal ratings for each component of the RDAI. The RDAI is a widely used tool in bronchiolitis research that evaluates wheezing and retractions on a scale from 0 to 17, with good interobserver reliability [7]. Scores of ≥6 and ≥15 indicate severe and very severe illness, respectively [8]. Clinical data were then abstracted from the chart following the visit. All data were managed using the Research Electronic Data Capture [9].

Sample size

We collected a convenience sample of infants with possible bronchiolitis who were present in the ED during the research assistants' observation period.

Measures

The primary outcome measure was the proportion of patients placed on continuous pulse oximetry during their ED stay. Secondary outcomes included disease severity, admission rates, return visits within 72 hours, use of supplemental oxygen, and investigations and interventions initiated in the ED. 

Analysis

We analyzed the data using descriptive statistics: proportions for categorical variables and medians and IQRs for continuous variables. We reported medians due to the small sample size and the presence of outliers that skewed the data.

Ethics approval was obtained from the IWK Health Research Ethics Board (Project 1027537, approved March 4, 2022).

## Results

During the study period, 18 patients met the inclusion criteria during an observation period (see Table 1). These patients ranged in age from 3 to 11 months, with a median age of 5 months (IQR 4, 7). The ED discharge diagnosis was a viral respiratory infection (upper respiratory tract infection or bronchiolitis) in 15 infants (83.3%), pneumonia in two infants (10.5%), and other in one infant (5.2%). Six patients (33.3%) were placed on continuous pulse oximetry during their ED stay. The median disease severity score, as measured by the RDAI, was 5.5 out of a possible 17 points (IQR 6.75) and appeared similar for infants who were continuously monitored (6.5 (IQR 5.3, 10.8)) and those who were not (4.5 (IQR 2, 9)). The average oxygen saturation of all infants was 96.6% with no clinically important difference between groups. The respiratory rate for infants who were continuously monitored was 61 (IQR 52.5, 62) and 44 (41.5, 48) for those who were not. The acceptable range of respiratory rate for infants in this age group is 30-60 breaths per minute; thus, despite a numerical difference between the groups, there is no important clinical difference between the groups. All infants placed on continuous pulse oximetry and eight out of 12 (66.7%) who were intermittently monitored received a CTAS score of 2. Overall, four infants (22.2%) had a chest radiograph, and four infants (22.2%) had bloodwork. In terms of interventions, three infants (16.7%) received antibiotics, four (22.2%) received bronchodilators, and one (5.6%) received systemic steroids. More patients in the continuous pulse oximetry group received investigations and interventions compared to the intermittent pulse oximetry group: chest radiograph and bloodwork in 50% versus 8.3%, antibiotics in 33.3% versus 8.3%, bronchodilators in 33.3% versus 16.7%, and steroids in 16.7% versus 0. Four patients (22.2%) were placed on supplemental oxygen and were subsequently admitted. All the infants placed on supplemental oxygen were in the continuous pulse oximetry group. One infant (8.3%) in the intermittent pulse oximetry group was admitted. Three infants (15.8%) had an unplanned return visit within 72 hours, and none was admitted to the hospital upon return. All the unplanned return visits occurred in the intermittent pulse oximetry group. No patient from our cohort was admitted to the intensive care unit. 

**Table 1:** Demographic and clinical characteristics RDAI: Respiratory Distress Assessment Index, CTAS: Canadian Triage and Acuity Scale, URTI: upper respiratory tract infection.

Variable, median (IQR)			
	Total (N = 18)	Continuous pulse oximetry (N = 6)	Intermittent pulse oximetry (N = 12)
Age (months)	5 (4, 7)	6.5 (4.5, 8.5)	5 (4, 6.3)
Respiratory rate at triage	48 (42.5, 59.5)	61 (52.5, 62)	44 (41.5, 48)
Oxygen saturation at triage	96.5 (95, 98.8)	95 (93.5, 95.8)	98 (96.8, 99)
RDAI	5.5 (2.25, 9)	6.5 (5.3, 10.8)	4.5 (2, 9)
Variable, frequency (%)			
Continuous pulse oximetry in ED	6 (33.3)	-	-
History of prematurity	2 (11.1)	2 (33.3)	0 (0)
CTAS			
2	15 (79.0)	6 (100)	8 (66.7)
3	4 (21.1)	0	4 (33.3)
Days of illness			
1-4	14 (77.8)	5 (83.3)	9 (75)
5-10	2 (11.1)	1 (16.7)	1 (8.3)
>10	2 (11.1)	0	2 (16.7)
Supplemental O2	4 (22.2)	4 (66.7)	0
Disposition			
Home	13 (72.2)	2 (33.3)	11 (92)
Inpatient	5 (27.8)	4 (66.7)	1 (8.3)
Discharge diagnosis			
Viral URTI	4 (21.1)	0	4 (33.3)
Bronchiolitis	11 (52.6)	5 (83.3)	6 (50)
Pneumonia	2 (10.5)	1 (16.7)	1 (8.3)
Other	1 (5.2)	0	1 (8.3)
Unplanned return visit (72 hours)	3 (15.8)	0	3 (25)
Chest X-ray	4 (22.2)	3 (50)	1 (8.3)
Bloodwork	4 (22.2)	3 (50)	1 (8.3)
Antibiotics	3 (16.7)	2 (33.3)	1 (8.3)
Bronchodilator	4 (22.2)	2 (33.3)	2 (16.7)
Steroids	1 (5.6)	1 (16.7)	0

## Discussion

We found that 33.3% of the observed infants were placed on continuous pulse oximetry. In an inpatient study, 46% of hospitalized infants with bronchiolitis who were admitted on room air were placed on continuous pulse oximetry [10]. The authors identified several risk factors for continuous pulse oximetry use, including younger age combined with a history of prematurity, time since weaning from supplemental oxygen, documented cyanosis or apnea, and nighttime shifts. In our cohort, we did not observe clear trends in clinical characteristics that led to the use of continuous monitoring. This suggests that the decision to place infants on continuous pulse oximetry may be influenced by provider (most often nurses) characteristics rather than by the objective clinical characteristics of the patients. In a multisite qualitative study of clinicians managing bronchiolitis in inpatient settings, multiple barriers to the de-implementation of continuous pulse oximetry were identified: perceived parental preference for monitoring, the challenge of caring for high- and low-acuity patients simultaneously, clinician discomfort, the “peace of mind” provided by monitoring, the ability to measure vital signs without waking the patients, and having less staff available at night [11]. It remains unclear whether ED nurses share similar experiences with continuous pulse oximetry use.

Previous literature suggests that infants with bronchiolitis can have frequent, prolonged, and profound oxygen desaturations throughout their illness [2,12]. However, in the absence of changes in respiratory effort, desaturations do not necessarily indicate that infants are sicker or require escalation of care. Desaturations that are undocumented due to intermittent pulse oximetry are likely clinically unimportant. However, when clinicians are aware of desaturations, infants are two times more likely to be admitted to the hospital [3]. In our cohort, 66.7% of infants in the continuous pulse oximetry group received supplemental oxygen and were subsequently admitted to the hospital. Furthermore, the continuous pulse oximetry group appeared to undergo more investigations and interventions than the intermittent pulse oximetry group, exceeding previously established achievable benchmarks for bronchiolitis care [13]: chest radiograph in 50% vs benchmark of 7.3%, bronchodilators in 33.3% vs benchmark of 8.8%, and steroids in 16.7% vs benchmark of 2.3%. Given the apparent similarity in clinical characteristics between the groups, this raises concerns about whether the increased interventions in the continuous pulse oximetry group were truly warranted or if the use of continuous pulse oximetry may have contributed to overuse in some cases.

There were several important limitations in interpreting our results. Despite planning for peak bronchiolitis season at our institution, the number of presentations for respiratory illness was lower than anticipated. Furthermore, while we demonstrated a feasible method for documenting continuous monitor use in infants with bronchiolitis, this resource-intensive approach required the in-person presence of research assistants, limiting the number of direct observations completed. This approach was modeled after a multisite study of infants with bronchiolitis in non-intensive care inpatient settings [14]. In institutions where electronic medical records are available with device integration to record vital signs from cardiorespiratory monitors, documentation of continuous pulse oximetry can be extracted directly for individual patients [15]. This method would allow for the analysis of all infants with bronchiolitis rather than relying on convenience samples. We also imposed strict inclusion criteria to avoid overlap with other causes of wheezing, which may have limited our ability to observe behaviors related to monitor use by excluding infants aged 12-24 months old. The small sample size also limited our ability to make comparisons between groups or examine risk factors for monitor use.

Future research should focus on understanding barriers and facilitators to reduce the use of continuous pulse oximetry in the ED. This will inform quality improvement initiatives by tailoring behavior change techniques accordingly. The development and implementation of pulse oximetry guidelines for infants with bronchiolitis in the ED must consider factors such as disease severity, nurse's motivations for using continuous pulse oximetry, and the potential harms and benefits of both continuous and intermittent pulse oximetry.

## Conclusions

We found that continuous pulse oximetry is used variably in infants with possible bronchiolitis in the ED and that investigations and interventions may have been influenced by its use. Our findings underscore the need for targeted education and the development of guidelines on the appropriate use of continuous pulse oximetry in infants with bronchiolitis in the ED, considering both potential benefits and harms.
